# Ezrin is a Major Regulator of Membrane Tension in Epithelial Cells

**DOI:** 10.1038/srep14700

**Published:** 2015-10-05

**Authors:** Bastian Rouven Brückner, Anna Pietuch, Stefan Nehls, Jan Rother, Andreas Janshoff

**Affiliations:** 1 Institute of Physical Chemistry, University of Goettingen, Tammannstr. 6, 37077 Goettingen

## Abstract

Plasma membrane tension is responsible for a variety of cellular functions such as motility, cell division, and endocytosis. Since membrane tension is dominated by the attachment of the actin cortex to the inner leaflet of the plasma membrane, we investigated the importance of ezrin, a major cross-linker of the membrane-cytoskeleton interface, for cellular mechanics of confluent MDCK II cells. For this purpose, we carried out ezrin depletion experiments and also enhanced the number of active ezrin molecules at the interface. Mechanical properties were assessed by force indentation experiments followed by membrane tether extraction. PIP_2_ micelles were injected into individual living cells to reinforce the linkage between plasma membrane and actin-cortex, while weakening of this connection was reached by ezrin siRNA and administration of the inhibitors neomycin and NSC 668394, respectively. We observed substantial stiffening of cells and an increase in membrane tension after addition of PIP_2_ micelles. In contrast, reduction of active ezrin led to a decrease of membrane tension accompanied by loss of excess surface area, increase in cortical tension, remodelling of actin cytoskeleton, and reduction of cell height. The data confirm the importance of the ezrin-mediated connection between plasma membrane and cortex for cellular mechanics and cell morphology.

Plasma membrane tension in eukaryotic cells is an important regulator of many cellular processes such as cell migration^1^,^2^, mitosis^3^, endocytosis^4^, exocytosis^5^, membrane repair^6^, osmoregulation^7^, and cell spreading^8^,^9^,^10^. In most of these processes cell shape changes generate considerable lateral stress in the plasma membrane compensated by surface area regulation to avoid membrane lysis^11^. The overall surface tension of the plasma membrane compiles contributions from the intrinsic surface tension of the lipid bilayer, adhesion, molecular connection between the plasma membrane and the underlying actin-cytoskeleton^12^,^13^,^14^, and an active contribution from the contractile actomyosin cortex^15^. It is indisputable that membrane tension of eukaryotic cells mainly originates from the linkage of the plasma membrane to the underlying cytoskeleton via protein linkers^13^,^16^. Evidence is accumulating that besides myosin I, membrane tension in eukaryotic cells is regulated by proteins of the ezrin-radixin-moesin (ERM) family^17^,^18^,^19^,^20^.

Other factors affecting plasma membrane tension include hydrostatic pressure across the membrane, and effects due to local membrane curvature associated with microvilli or invaginations such as caveolae^21^. The cell responds to changes in tension by adjusting its overall surface area, for instance, by activation of mechanosensitive ion channels that govern the rates of exo- and endocytosis^22^ or recruiting excess membrane from membrane infoldings or protrusions in order to avoid lysis of the membrane. Due to its liquid crystalline nature the plasma membrane cannot dilate beyond a maximum of about 2–3% resulting in lysis of the bilayer structure^23^,^24^,^25^. Typical membrane tensions are, however, 100- to 1000-fold lower than the lysis tension of a lipid bilayer^4^,^26^,^27^. Even lower tension is only found if the cytoskeleton is compromised or phosphatidylinositol 4,5-bisphosphate (PIP_2_) is depleted from the plasma membrane^16^. This implies that mammalian cells use membrane-remodeling mechanisms to buffer tension changes such as endocytosis and exocytosis but also release of membrane material from reservoirs in the plasma membrane. In essence, tension-driven surface area regulation is realised through supply of excess plasma membrane area to accommodate high tension and a reduction of membrane area if the tension is low. Along these lines, Nassoy and coworkers reported that cells respond to mechanical stress by sacrificing caveolae structures compensating an increase in tension^28^. Early work of Raucher and Sheetz also showed that elevated tension in conjunction with decreased endocytosis is a general phenomenon in mitotic cells^3^.

The goal of the present study is to understand how the linkage between the plasma membrane and the actomyosin cortex impacts cellular morphology and mechanics through regulation of the membrane tension exerted by the presence of activated ezrin. Ezrin belongs to the ERM protein family whose primary function is mediating a dynamic linkage between the plasma membrane and cortical actin located just below the membrane^29^. One of the most fundamental aspects of ERM protein functions is their ability to regulate this connection by switching between an active and an inactive (dormant) conformation. In the active conformation, the N-terminal region (FERM domain) binds to plasma membrane lipids and cytoplasmic tails of transmembrane proteins, while the C-terminal region binds to F-actin. By contrast, in the dormant conformation, those two regions are associated to each other and therefore not accessible by actin filaments and plasma membrane binding sites. This conformational switch between dormant and active form is initiated and sustained by binding to PIP_2_ located in the plasma membrane and phosphorylation of a threonine residue (Thr-567), which is the target for phosphorylation by Rho-kinase^30^,^31^, protein kinase C*θ*^32^, and protein kinase C*α*^33^.

In this study we used Madin-Darby canine kidney cells (MDCK II) to investigate how polar epithelial cells respond to an increased PIP_2_ level or alternatively, ezrin depletion. To increase the PIP_2_ level in the inner membrane leaflet we injected PIP_2_ micelles into individual cells paired with indentation experiments and subsequent tether pulling using an atomic force microscope (AFM). Ezrin depletion was accomplished by microinjection of neomycin or exposure of ezrin inhibitor NSC 668394 to the cells^34^. Alternatively, short interference RNA (siRNA) was employed to abolish ezrin expression^35^. Regardless of how ezrin is removed from the membrane-cortex interface, membrane tension decreases, cells sacrifice excess surface area, and cells contract.

## Results

Starting point for this work was the finding that the interaction between PIP_2_ activated ezrin and actin filaments *in vitro* is highly dynamic mirrored in ezrin-actin off-rates on the order of seconds^17^,^36^,^37^. Tether pulling of PIP_2_-microinjected MDCK II cells in comparison to untreated cells revealed that membrane tension is mainly governed by the presence of active ezrin^17^. This finding was urging the question to what extent this membrane-cortex interface is responsible for the mechanical properties of living epithelial cells and how tension is used by epithelial cells for mechanotransduction. The aim of the present study was therefore to draw a comprehensive picture of the mechanical response of cells after interference with membrane-cytoskeleton attachment sites. For this purpose, we, on the one hand, reinforced this connection via microinjection of PIP_2_ micelles into single epithelial cells. On the other hand, we weakened the binding using a variety of methods to minimise secondary effects. First we blocked the PIP_2_ binding site in single cells of a confluent layer via microinjection of neomycin. In addition, to examine the behaviour of a full cell layer of ezrin lacking cells, we used the ezrin inhibitor NSC 668394 as a chemical inhibitor and short interference RNA (siRNA) for ezrin knock-down.

The mechanical experiments were all carried out using confluent MDCK II cells. Force indentation curves were described by a tension model assuming that the restoring force to deformation of the cell originates only from pre-stress and area dilatation of the plasma membrane and cortex under a constant enclosed volume. Generally, the non-deformed shape of the cell needs to be known using an appropriate parameterisation that allows computing the deformed contour of the cell. Force balance consistent with Young Laplace’s law provides the restoring force only as a function of area dilatation and pre-stress. The model assumes a constant isotropic tension *T*:


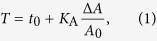


in which the pre-stress or overall tension *t*_0_ combines linearly with a term describing the stretching or area dilatation of the bilayer at large strain assuming constant volume. Δ*A* denotes the change in surface area related to the initial area of the cell surface *A*_0_ prior to indentation. *K*_A_ is the area compressibility modulus of the plasma membrane/cortex. At small strain the pre-stress or overall tension *t*_0_ dominates the elastic response, while larger deformation inevitably results in stretching of the shell limited by the inextensibility of the plasma membrane. Pre-stress *t*_0_ is dominated by an active cortical tension *t*_act_ of the actomyosin cortex with an additional contribution from the membrane tension *t*_t_ that itself arises mainly due to the presence of cytoskeleton-membrane attachment sites.


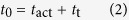


Since the AFM tip is functionalised with a lectin, retraction of the AFM tip frequently generates membrane nanotubes (tethers). The specific tether force *F*_tether_ is a measure of the connection strength between the plasma membrane and underlying cytoskeleton. The force plateau (*F*_tether_) of a fully established membrane tether formed upon retraction of the cantilever allows computing the membrane tension *t*_t_ if the bending modulus is known^13^,^38^. Here, we refrain from computing *t*_t_ but instead provide the forces to extract the nanotubes.

Excess surface area stored in wrinkles and folds of the plasma membrane reduces *K*_A_. Therefore, we usually replace *K*_A_ by an apparent area compressibility modulus 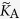 accounting for the excess surface area^27^. This procedure allows us to gather site-specific mechanic information about membrane tension, cortical tension and available surface area.

**Figure 1:**
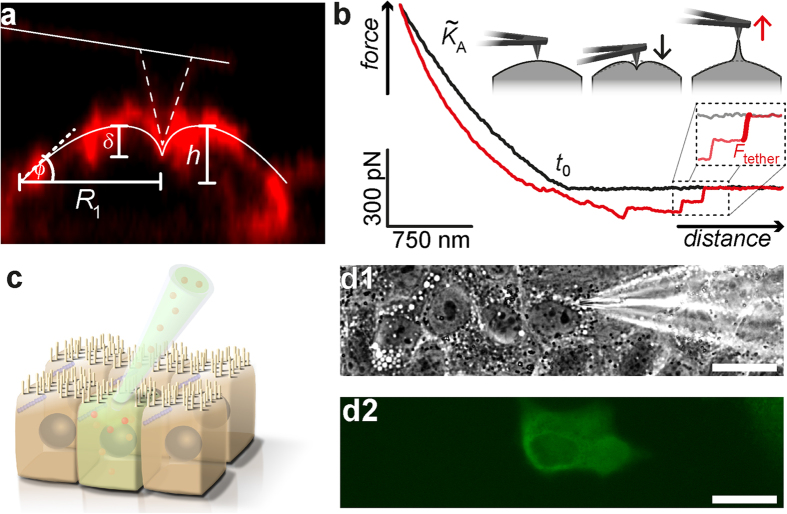
Schemes and data illustrating AFM experiments and microinjection. (**a**) Confocal image (*xz* plane) of the plasma membrane of a MDCK II cell stained with CellMask™ Orange. The cell is indented with an AFM probe up to a force of 1 nN. The geometry and parameterisation of the apical cap are shown. Parameters: base radius of the apical cap (*R*_1_), height of the apical cap (*h*), contact angle 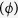 and indentation depth 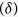. (**b**) Representative force—indentation (black) and force—retraction (red) curve. The inset shows how the tether rupture force *F*_tether_ is determined. (**c**) PIP_2_ micelles were microinjected into a single MDCK II cell of a confluent monolayer. A co-injected fluorescent dye (green) serves as marker. (**d1**) Phase contrast image showing confluent MDCK II cells subject to injection. The micropipette is positioned to inject a single cell. (**d2**) The successfully injected cell can be detected via fluorescence microscopy. (Scale bar: 10 μm).

Figure 1 illustrates how the experiments were performed and how the relevant parameters such as overall tension *t*_0_, apparent area compressibility modulus 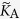 and tether forces *F*_tether_ are extracted from the force curves. We assume that the apical part of MDCK II cells adopts a spherical cap and that the volume of the cell is conserved. Moreover, we restrict recruitable membrane area to the area of the apical cap fixed by tight junctions and the adherens belt. Consequently, we compute the geometry of the indented spherical cap as a function of indentation depth. These conditions generate essentially non-linear force-indentation curves according to membrane theory^27^,^39^,^40^. By assuming that tension is the only source of restoring force we can numerically reconstruct the force indentation curve with only two parameters, the pre-stress or overall tension *t*_0_ comprising active cortical tension plus membrane tension and the apparent area compressibility modulus 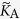 (Fig. 1a,b)^27^. In principle, the maximal area compressibility modulus is limited to the value of the spread-out pure lipid bilayer (0.1–1.0 N/m)^41^ devoid of membrane reservoirs. At larger indentation depth the force curves adopt a non-linear shape due to stretching of the shell at larger strain (Fig. 1b). Lower values of 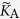 than of a lipid bilayer are attributed to excess membrane area stored in reservoirs such as folds, caveloae, microvilli or wrinkles.

### Increasing the Number of PIP_2_ binding sites increases cell stiffness

**Figure 2:**
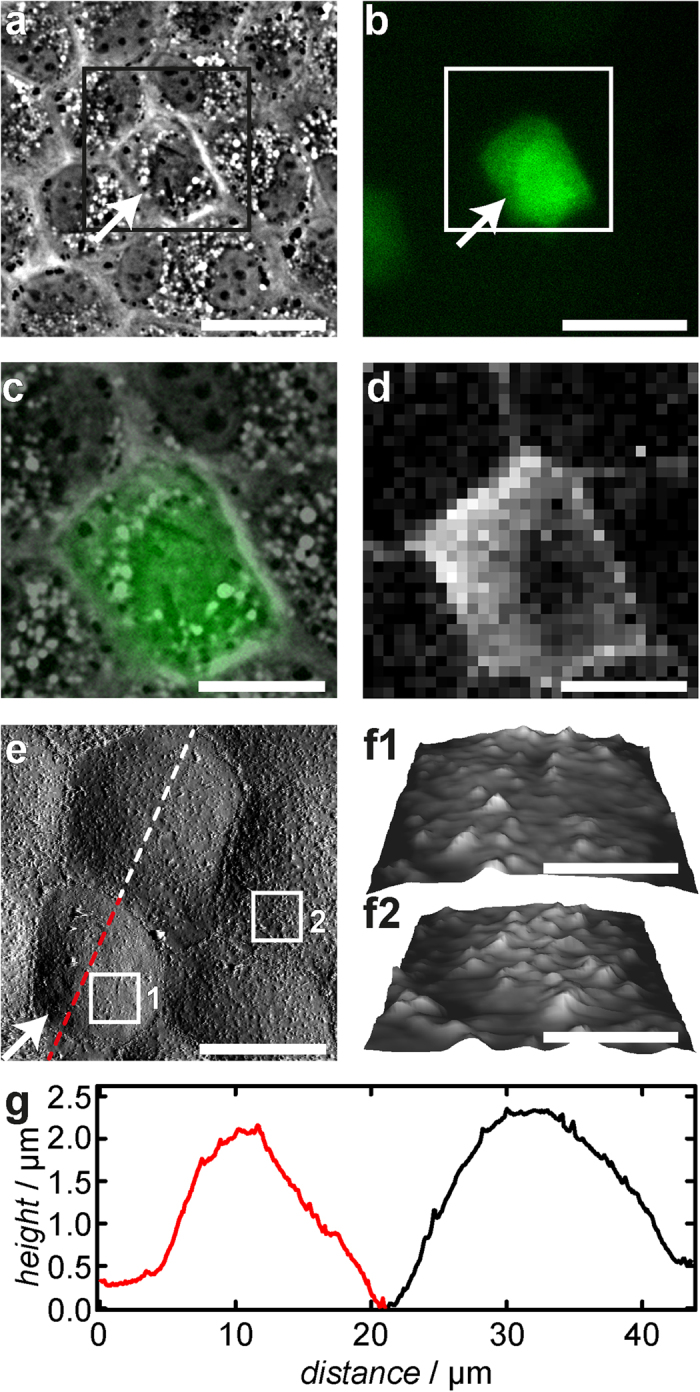
Microinjection of PIP_2_ micelles into confluent MDCK II cells. (**a**) Phase contrast image of MDCK II cells, 3 h after microinjection of PIP_2_ micelles. (**b**) Corresponding fluorescence micrograph of the same area shown in (**a**). FITC-dextran (green) was co-injected to identify successfully modified cells. The manipulated cell is marked with an arrow. (**c**) Overlay of the boxed regions from (**a**,**b**). (**d**) Force map of the region shown in (**c**), recorded 3 h after microinjection of PIP_2_ micelles. Every pixel represents one single force-distance curve. The gray scale corresponds to the slope of the curve representing essentially the area compressibility modulus of the cell. (**e**) AFM deflection image. An arrow marks the microinjected cell. (**f**) Three dimensional AFM height images of the corresponding boxes shown in (**e**). (**g**) Height profile along the red/white dotted line drawn in (**e**). (Scale bar: (**a**,**b**,**e**): 30 μm, (**c**,**d**): 15 μm, (**f**): 1 μm).

**Figure 3:**
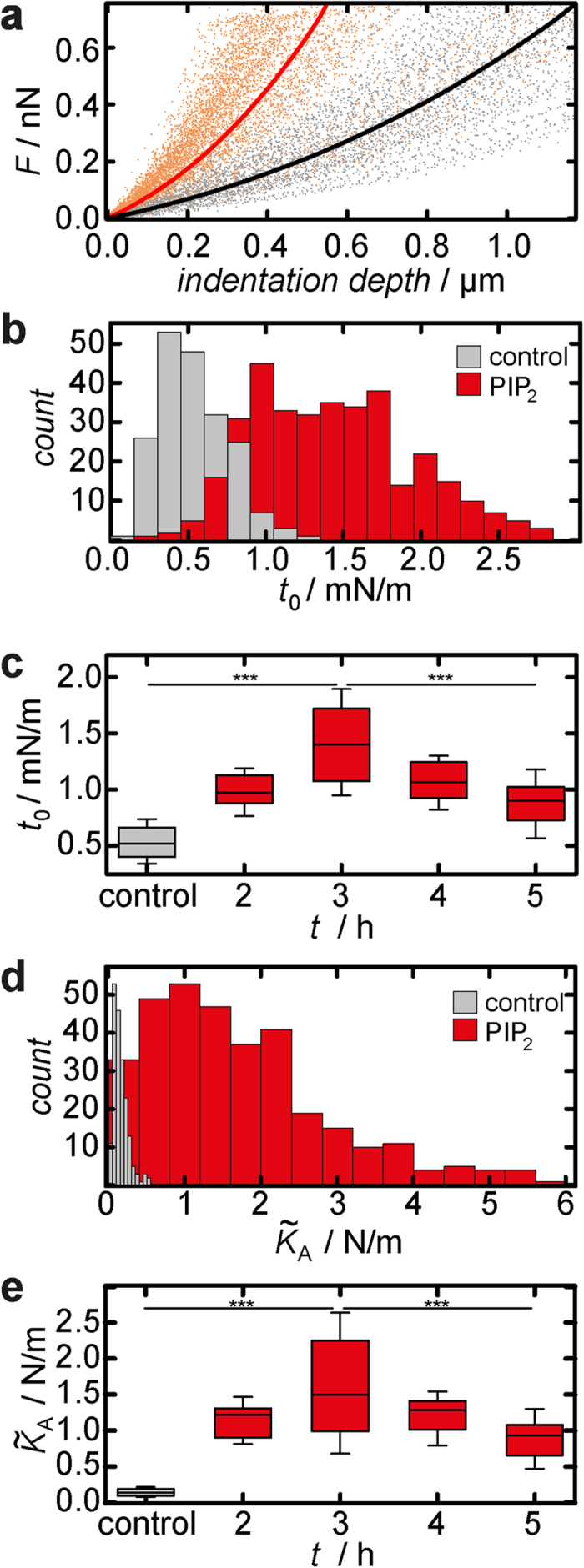
Cellular mechanics of MDCK II cells after PIP_2_ microinjection. (**a**) Averaged indentation curves for PIP_2_ microinjected (red) and control cells (black). (**b,c**) Overall tension 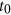. (**d**,**e**) Apparent area compressibility modulus 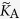. (**b**, **d**) Histograms obtained from analysis of force indentation curves on control cells (grey) and 3 h after injection of PIP_2_ (red). (**c**,**e**) show 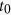 and 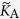 as a function of elapsed time after PIP_2_ injection. Box plots extend from the 30th to the 70th percentile, whiskers from the 20th to the 80th. Grey bars in the histograms and box plots refer to data obtained from untreated cells, red ones represent PIP_2_ microinjected cells. (**b**,**c**) *n* = 196 (control), 132 (2 h), 106 (3 h), 101 (4 h), 71 (5 h) analysed force distance curves. (**d**,**e**) *n* = 196 (control), 132 (2 h), 102 (3 h), 101 (4 h), 71 (5 h) analysed force distance curves. *n* > 5 (control), *n* = 3 (PIP_2_) analysed cells. Asterisks indicate a statistical difference (***p < 0.001, Wilcoxon rank sum test).

PIP_2_ micelles were microinjected into single MDCK II cells, while adjacent cells remain unaffected and serve as an intrinsic control. A fluorescent dye (fluorescein isothiocyanate-dextran (FITC-dextran), green) was co-injected to identify cells after microinjection (Fig. 1c,d). Figure 2a–c shows optical micrographs (c: overlay of phase contrast and fluorescence) of a confluent MDCK II cell monolayer in which a single cell has been successfully microinjected with PIP_2_ micelles (green). Plasma membrane integrity was verified 3 h after microinjection using propidium iodide. As shown recently, microinjection of PIP_2_ recruits more ezrin to the interface between plasma membrane and cortex thereby a triggering redistribution of F-actin^17^. The reinforcement of the connection between plasma membrane and cytoskeleton is reflected in larger tether force *F*_tether_ due to an increased membrane tension *t*_t_^13^,^17^,^18^. Now we asked, how remodeling of the actin cytoskeleton, provoked by increased recruitment of ezrin to the plasma membrane, affects cellular mechanics and morphology. For this purpose we used AFM imaging paired with force volume measurements and microinjection of PIP_2_ (Fig. 2d–g). Importantly, the height and the radius of the cells are not altered after microinjection of PIP_2_ micelles (Fig. 2g). To examine the mechanical response to indentation more closely, we recorded time-resolved force indentation maps with the AFM (Fig. 2d and Fig. 3). Averaged force indentation curves carried out either on microinjected cells (red) or adjacent control cell (black) are shown in Fig. 3a. Even without applying a mechanical model to describe the elasticity of the cell in terms of modules it is clear that the cells become significantly stiffer with increasing PIP_2_ level. To assess the overall tension *t*_0_ and the apparent area compressibility modulus 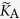 from the indentation curves we followed a procedure recently introduced by Pietuch *et al.*^27^. The shape of the spherical cap was reconstructed from the base radius *R*_1_ and contact angle *ϕ* measured by AFM imaging. The average base radius of the apical cap (Fig. 1a) was found to be *R*_1_ = 12 μm and the average contact angle *ϕ* *=* 20°. This knowledge allows computing force-indentation curves assuming constant volume during indentation. As expected from the averaged force curves both parameters, *t*_0_ and 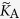 increase after administration of PIP_2_ (Fig. 3b–e). *t*_0_ dominates at low penetration depth, while 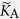 governs the mechanical response at large strain. Maximal change of the mechanical parameters after administration of PIP_2_ was found 3 h after microinjection. For instance, we found that overall tension *t*_0_ increases from (0.52 ± 0.02) mN/m (median ± SEM) to (1.4 ± 0.3) mN/m in the first 3 h after microinjection of PIP_2_ (Fig. 3b/c). The increase in overall tension is attributed to a larger number of contact sites between F-actin and plasma membrane responsible for a larger membrane tension as well as a reinforced cortex producing a larger active cortex tension due to the contractile nature of the actomyosin network resulting in a larger *t*_0_. However, 5 h after treatment the tension drops back to (0.90 ± 0.04) mN/m (Fig. 3c) indicative of tension homeostasis. Along similar lines, the apparent area compressibility modulus 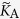 rises from (0.14 ± 0.1) N/m to (1.5 ± 0.09) N/m in the first 3 h after PIP_2_ injection (Fig. 3d/e). Subsequently, a down regulation to (0.93 ± 0.05) N/m (after 5 h) sets in (Fig. 3e). An increase in 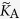 can be rationalised by a reduction of membrane reservoirs or change of overall cell geometry. Besides, a thicker cortex would also lead to a larger apparent area compressibility modulus. Apart from the number of wrinkles and folds the connection strength between the plasma membrane and underlying cytoskeleton might also limit the number of accessible membrane reservoirs.

### Weakening of the plasma membrane-cytoskeleton attachment leads to substantial changes in cell morphology and cell mechanics

**Figure 4:**
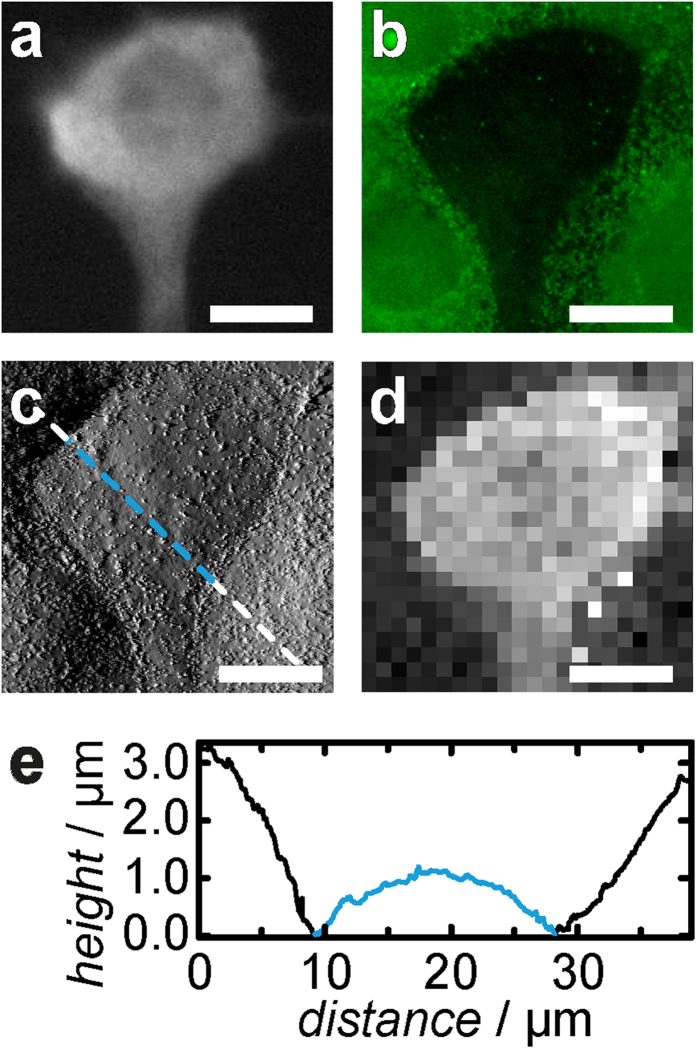
Effects of neomycin on confluent MDCK II cells. (**a**) MDCK II cell after microinjection of neomycin. FITC dextran was co-injected to detect the microinjected cell after 3 h of administration. (**b**) Fluorescence micrograph showing fluorescently labelled ezrin (green) using ezrin mouse IgG primary and Alexa Fluor 488 labelled goat anti-mouse secondary antibody. No ezrin could be detected in the neomycin microinjected cell. (**c**) AFM deflection image corresponding to (**a**,**b**). (**d**) Force map of the region shown in (**a**–**c**), recorded 3 h after microinjection of neomycin. Every pixel represents one single force-distance curve. The grey scale corresponds to the slope of the curve representing approximately the area compressibility modulus of the cell. (**e**) Height profile along the white/blue dotted line in (**c**) envisioning topographical changes due to neomycin microinjection. (Scale bar: 10 μm).

**Figure 5:**
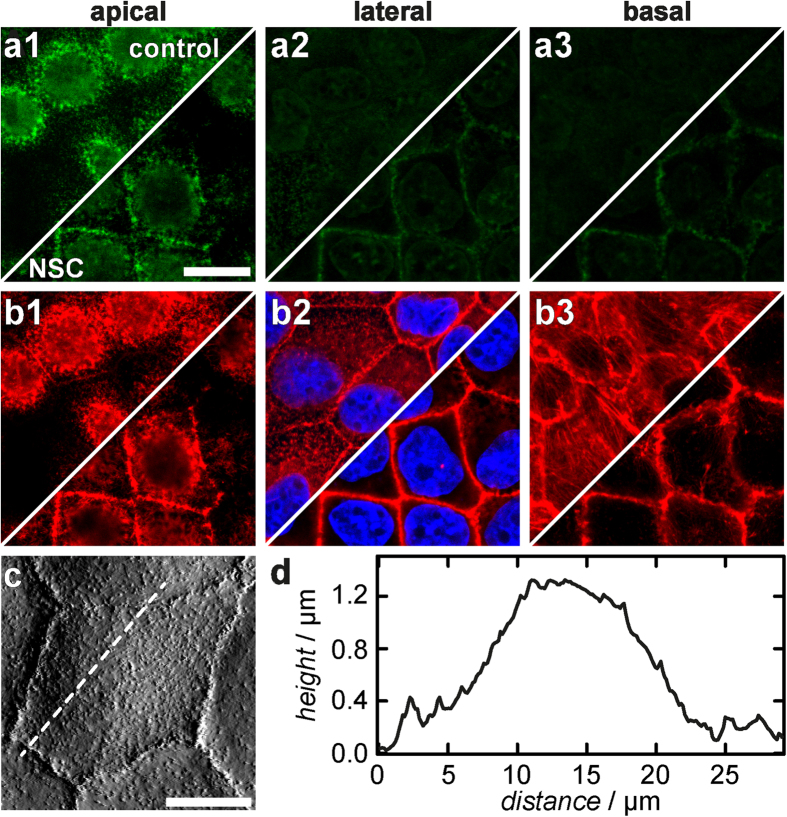
Effects of NSC 668394 on confluent MDCK II cells. (**a**,**b**) Confocal images showing changes of the ezrin (**a**) and F-actin (**b**) distribution on the apical (**1**), lateral (**2**), and basal (**3**) site, respectively. (**c**) AFM deflection image of a NSC 668394 treated MDCK II cell. (**d**) Height profile along the white dotted line in (**c**). (Scale bar: (**a**,**b**): 15 μm, (**c**): 10 μm).

**Figure 6:**
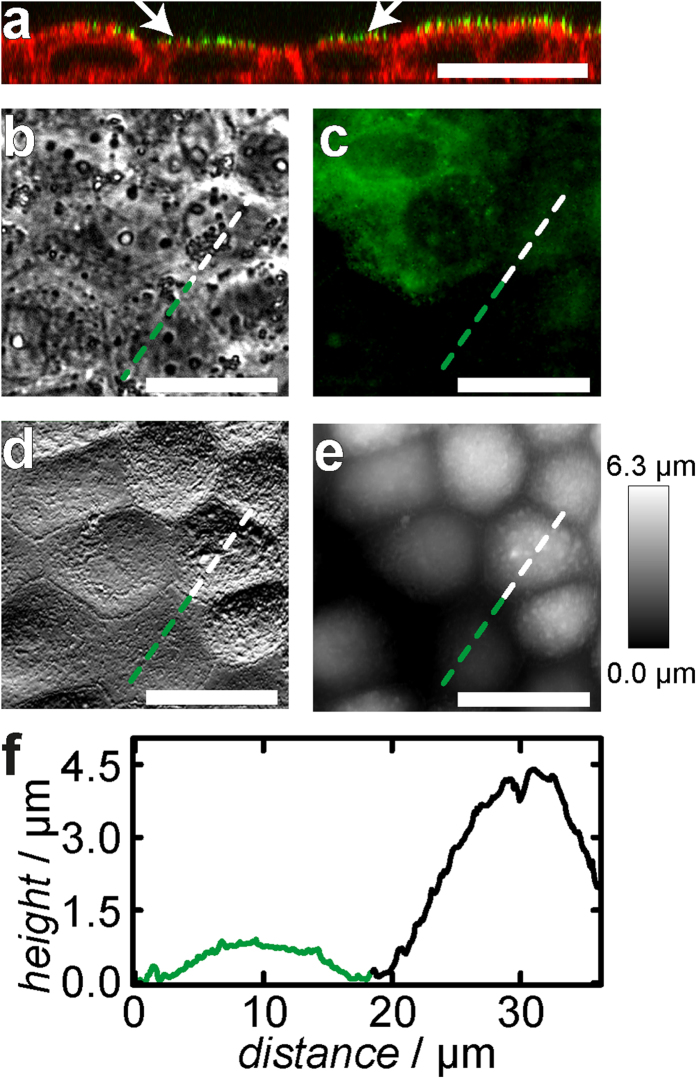
Topography of MDCK II cells after ezrin knock-down via siRNA. (**a**) Confocal fluorescence micrograph of the *xz* plane of a confluent cell layer. The plasma membrane is stained with PKH67 Green Fluorescent Cell Linker, F-actin is labeled with Alexa Fluor 546-phalloidin. Cells without ezrin (arrows) were found to be reduced in height compared with not successfully transfected cells. (**b**) Phase contrast image. (**c**) Corresponding fluorescence micrograph showing the ezrin distribution. Ezrin is stained with ezrin mouse IgG primary and Alexa Fluor 488 labeled goat anti-mouse IgG secondary antibody. Some cells (green) were not successfully transfected with siRNA and serve as a control. (**d**) AFM deflection and height image (**e**) of the same spot shown in (**b**,**c**). (**f**) Height profile along the green/white dotted line. Cells lacking ezrin are substantially flattened. (Scale bar: 20 μm).

**Figure 7:**
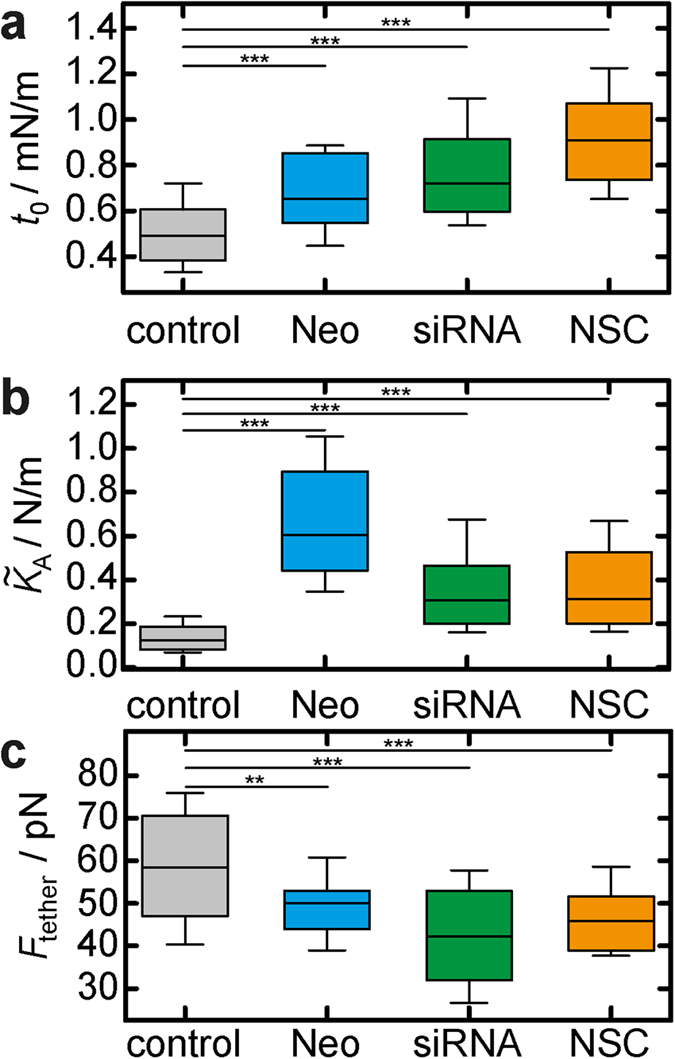
Mechanical investigation of MDCK II cells after ezrin depletion. (**a**) Overall tension 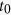 obtained from indentation experiments. (**b**) Apparent area compressibility modulus 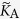. (**c**) Tether rupture for**c**e 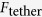 obtained from tether pulling experiments. Box plots extend from the 30th to the 70th percentile, whisker from the 20th to the 80th. Grey boxes represent results from untreated cells, blue ones are from neomycin microinjection experiments, green ones represent cells after siRNA treatment, orange ones show results from cells exposed to ezrin inhibitor NSC 668394. (**a**) *n* = 389 (control), 38 (neomycin), 129 (siRNA), 317 (NSC 668394) analysed force distance curves. (**b**) *n* = 374 (control), 38 (neomycin), 129 (siRNA), 313 (NSC 668394) analysed force distance curves. (**c**) *n* = 257 (control), 37 (neomycin), 85 (siRNA), 39 (NSC 668394) analysed force distance curves. *n* > 20 (control), *n* = 2 (neomycin), *n* > 20 (siRNA), *n* > 15 (NSC 668394) analysed cells. Asterisks indicate a statistical difference (**p < 0.01, ***p < 0.001, Wilcoxon rank sum test).

Enforcing the connection between plasma membrane and underlying actin cortex by adding PIP_2_ to the inner leaflet of the plasma membrane results in increased membrane tension, overall tension, and actin remodelling. In order to address the reverse effect, a reduction of the actin-membrane attachment sites, we performed three different experiments: masking of PIP_2_ binding sites for ezrin through microinjection of the antibiotic neomycin (Fig. 4), inhibition of ezrin by adding of NSC 668394 (Fig. 5) and knock-down of ezrin expression by using siRNA (Fig. 6). Neomycin binds to PIP_2_ and thereby uncouples the membrane from the cytoskeleton^42^. After microinjection of the antibiotic (Fig. 4a), the cell showed no signal for the ezrin immunostaining (Fig. 4b). AFM images revealed flattening of the cell compared to adjacent neighbours by approximately 1 μm (Fig. 4c/e). Tether pulling from the plasma membrane was perfomed with AFM tips functionalised with Concanavalin A to enable a strong binding to the plasma membrane during retraction of the cantilever. Based on the results described above we expected a significant lower tether rupture force *F*_tether_ due to the loss of attachment sites for ezrin. After microinjection of neomycin *F*_tether_ drops slightly from (58.5 ± 1.2) pN to (50.0 ± 1.8) pN (Fig. 7c, Supplementary Fig. S4), indicating a weakened connection between plasma membrane and underlying cytoskeleton. Notably, controls shown in figure 7 comprise all cells of this study. Force indentation curves were modeled using an average base radius of *R*_1_ = 10 μm and a contact angle of *ϕ* = 12° to account for the altered cell morphology, i.e. flattening of the apical cap (Fig. 4e). 3 h after microinjection of neomycin we found an increasing overall tension (*t*_0_ = (0.65 ± 0.04) mN/m, control: *t*_0_ = (0.49 ± 0.01) mN/m) (Fig. 7a, Supplementary Fig. S2). The apparent area compressibility modulus was also found to be enlarged to 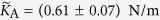 (control: 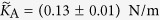) (Fig. 7b, Supplementary Fig. S3) indicating a loss of excess membrane area or detection of other contributions that stiffens the cell.

To ensure that the mechanical response is caused indeed by altering the amount of PIP_2_ or blocking this ezrin binding site via neomycin and not only due to the physical stress applied on the cell during microinjection, FITC dextran in the absence of PIP_2_ micelles or neomycin was injected in single cells. Again indentation experiments were carried out 3 h after microinjection. We found no significant changes in the mechanical behaviour compared to untreated cells (Enforcing the connection between plasma membrane and underlying actin cortex by adding PIP_2_ to the inner leaflet of the plasma membrane results in increased membrane tension, overall tension, and actin remodelling. In order to address the reverse effect, a reduction of the actin-membrane attachment sites, we performed three different experiments: masking of PIP_2_ binding sites for ezrin through microinjection of the antibiotic neomycin (Fig. 4), inhibition of ezrin by adding of NSC 668394 (Fig. 5) and knock-down of ezrin expression by using siRNA (Fig. 6). Neomycin binds to PIP_2_ and thereby uncouples the membrane from the cytoskeleton^42^. After microinjection of the antibiotic (Fig. 4a), the cell showed no signal for the ezrin immunostaining (Fig. 4b). AFM images revealed flattening of the cell compared to adjacent neighbours by approximately 1 μm (Fig. 4c/e). Tether pulling from the plasma membrane was perfomed with AFM tips functionalised with Concanavalin A to enable a strong binding to the plasma membrane during retraction of the cantilever. Based on the results described above we expected a significant lower tether rupture force *F*_tether_ due to the loss of attachment sites for ezrin. After microinjection of neomycin *F*_tether_ drops slightly from (58.5 ± 1.2) pN to (50.0 ± 1.8) pN (Fig. 7c, Supplementary Fig. S4), indicating a weakened connection between plasma membrane and underlying cytoskeleton. Notably, controls shown in figure 7 comprise all cells of this study. Force indentation curves were modeled using an average base radius of *R*_1_ = 10 μm and a contact angle of *ϕ* = 12° to account for the altered cell morphology, i.e. flattening of the apical cap (Fig. 4e). 3 h after microinjection of neomycin we found an increasing overall tension (*t*_0_ = (0.65 ± 0.04) mN/m, control: *t*_0_ = (0.49 ± 0.01) mN/m) (Fig. 7a, Supplementary Fig. S2). The apparent area compressibility modulus was also found to be enlarged to 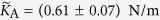 (control: 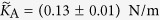) (Fig. 7b, Supplementary Fig. S3) indicating a loss of excess membrane area or detection of other contributions that stiffens the cell.

Enforcing the connection between plasma membrane and underlying actin cortex by adding PIP_2_ to the inner leaflet of the plasma membrane results in increased membrane tension, overall tension, and actin remodelling. In order to address the reverse effect, a reduction of the actin-membrane attachment sites, we performed three different experiments: masking of PIP_2_ binding sites for ezrin through microinjection of the antibiotic neomycin (Fig. 4), inhibition of ezrin by adding of NSC 668394 (Fig. 5) and knock-down of ezrin expression by using siRNA (Fig. 6). Neomycin binds to PIP_2_ and thereby uncouples the membrane from the cytoskeleton^42^. After microinjection of the antibiotic (Fig. 4a), the cell showed no signal for the ezrin immunostaining (Fig. 4b). AFM images revealed flattening of the cell compared to adjacent neighbours by approximately 1 μm (Fig. 4c/e). Tether pulling from the plasma membrane was perfomed with AFM tips functionalised with Concanavalin A to enable a strong binding to the plasma membrane during retraction of the cantilever. Based on the results described above we expected a significant lower tether rupture force *F*_tether_ due to the loss of attachment sites for ezrin. After microinjection of neomycin *F*_tether_ drops slightly from (58.5 ± 1.2) pN to (50.0 ± 1.8) pN (Fig. 7c, Supplementary Fig. S4), indicating a weakened connection between plasma membrane and underlying cytoskeleton. Notably, controls shown in figure 7 comprise all cells of this study. Force indentation curves were modeled using an average base radius of *R*_1_ = 10 μm and a contact angle of *ϕ* = 12° to account for the altered cell morphology, i.e. flattening of the apical cap (Fig. 4e). 3 h after microinjection of neomycin we found an increasing overall tension (*t*_0_ = (0.65 ± 0.04) mN/m, control: *t*_0_ = (0.49 ± 0.01) mN/m) (Fig. 7a, Supplementary Fig. S2). The apparent area compressibility modulus was also found to be enlarged to 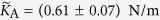 (control: 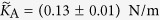) (Fig. 7b, Supplementary Fig. S3) indicating a loss of excess membrane area or detection of other contributions that stiffens the cell. Supplementary Fig. S6).

Besides neomycin we also used the small molecule NSC 668394 to inhibit phosphorylation of the conserved threonine residue Thr-567 that is important for binding of ezrin to the F-actin cytoskeleton^34^. After exposure of NSC 668394 for 3 h to the cells CLSM images showed significant changes in the ezrin distribution of treated cells. Ezrin was no longer found as point-like features at the apical side, but displayed a more dispersed distribution. The protein accumulated strongly at cell-cell boundaries (Fig. 5a). We also investigated changes of the F-actin organisation (Fig. 5b). At the apical side we found actin structures colocalising with the ezrin distribution. Sharp dots, representing microvillar structures, disappear (Fig. 5b, panel 1), while the staining becomes blurrier. Also, F-actin at the cell-cell boundaries on the lateral side of the cells is more pronounced after NSC 668394 treatment (Fig. 5b, panel 2). Interestingly, basal stress fibres, characteristic for polarised MDCK II cells, also vanish in response to NSC 668394 administration (Fig. 5b, panel 3).

We also investigated the topography of cells treated with NSC 668394. The height of the apical cap is reduced, comparable with the morphological changes of cells exposed to neomycin (Fig. 5c/e). The contractile ring at the cell-cell boundaries is more pronounced in AFM images—an indication, that the apical plasma membrane is lowered, whereas a structure-generating distribution of F-actin at the lateral site is maintained or even more pronounced (*vide supra*).

Again, force retraction curves were analysed to determine the tether rupture force *F*_tether_ reporting on the membrane tension. *F*_tether_ dropped substantially from (58.5 ± 1.2) pN for untreated cells to (45.8 ± 1.4) pN 3 h after drug administration (Fig. 7c, for histogram see Supplementary Fig. S4). Force indentation curves were modeled using the same geometric parameters as used for force indentation curves recorded on neomycin-microinjected cells. The overall tension *t*_0_ increased from *t*_0_ = (0.49 ± 0.01) mN/m (control) to (0.91 ± 0.03) mN/m (Fig. 7a, for histogram see Supplementary Fig. S2). The apparent area compressibility modulus increased from 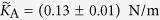 to a 2.5-fold higher value 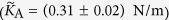 (Fig. 7b, for histogram see Supplementary Fig. S3). The possible impact of DMSO used as a solvent for the drug on cellular morphology or cellular mechanics, was ruled out in control experiments (Supplementary Fig. S7).

The most reliable approach to destroy the plasma membrane cytoskeleton connection without side effects was the application of genetic techniques for ezrin depletion. Figure 6 shows that confluent MDCK II cells have lost most of the cross linker ezrin after treatment with appropriate siRNA. Western blotting also demonstrates a significantly lower ezrin signal for cells treated with siRNA compared to control cells (see Supplementary Fig. S1a/d). We also analysed the actin distribution finding no obvious changes (see Supplementary Fig. S1b). However, after analysing cell morphology using AFM and CLSM imaging we found that again the apical cap is flattened down to approximately 0.8 μm (Fig. 6a/e,f). Figure 6 shows a spot on the culture dish in which successfully transfected cells coexist with barely transfected ones to illustrate the impact of ezrin knock-down on cell morphology within a single image. The base radius of the apical cap is only slightly affected by the absence of ezrin in the cell. Furthermore, the contractile actin ring which is proposed to serve for tension generation^27^ in connection with cell-cell contacts is also still visible (Fig. 6d).

From force retraction curves we obtain a lowered tether rupture force of (42.3 ± 1.6) pN (control: *F*_tether_ = (58.5 ± 1.2) pN). The mechanical parameters *t*_0_ and 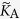 were obtained from fitting of force indentation curves using the same geometric parameters as used for neomycin and NSC 668394 experiments. We found an increase in membrane tension after ezrin silencing (*t*_0_ = (0.72 ± 0.04) mN/m, control: *t*_0_ = (0.49 ± 0.01) mN/m). 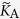 also increases from *t*_0_ = (0.13 ± 0.01) N/m to (0.31 ± 0.04) N/m for siRNA treated cells (Fig. 7, for histograms see Supplementary Fig. S2–4).

To ensure that the changes in the mechanical behaviour originate from the ezrin knock-down and not from the transfection procedure, we applied non-targeting siRNA in the same way as ezrin siRNA. No significant changes in the mechanical behaviour were observed (see Supplementary Fig. S7).

Figure 7 summarises the impact of weakening the plasma membrane-cortex interface on the various mechanical parameters. While the overall tension *t*_0_ increases in all cases, membrane tension or better yet the tether force drops as expected. This means that cortical tension due to a higher contractility increases considerably after ezrin depletion at this interface. The cells subject to ezrin depletion flatten substantially and at the same time the cells stiffen at larger strain. If we interpret this finding in terms of diminished excess surface area of the apical cell membrane in response to ezrin depletion we should expect a decrease in membrane capacitance. Therefore, we performed impedance measurements of confluent MDCK II cells cultured on microelectrodes using electric cell-substrate impedance sensing^43^. We found that indeed the membrane capacitance *C*_m_ capturing the entire area of the plasma membrane decreases after administration of NSC 668394 by 16% (see Supplementary Fig. S5). Interestingly, also *R*_b_ representing the transepithelial resistance drops after 3 h of incubation with NSC 668394. This can be explained by the fact that ezrin connects actin also to the lateral membrane. Weakening of this connection results in opening of tight junctions^44^.

## Discussion

We investigate the cellular mechanics of confluent epithelial cells in the context of the interface formed between the actin cortex and the plasma membrane. By injection of an excess of PIP_2_ we fortified the ezrin-mediated connection between plasma membrane and F-actin provoking an increase in the number of bonds formed by membrane-associated ezrin. Consequently, overall tension of the cortex/membrane shell increased as expected but also excess membrane area was removed. We found that not only membrane tension increased but also the contractility of the cortex presumably due to actin remodelled leading to a larger active cortical tension. Thus the mechanical behaviour of the entire cell was altered tremendously and the cell strives to reduce tension and restores the accessible membrane reservoirs, which took a few hours. One can, however, ask the question, if the injected amount of only a few femtoliter of PIP_2_ solution is sufficient to enhance the PIP_2_ level in the way we found. PIP_2_ regulates phospholipase D positively, which produces phosphatic acid, a regulator for PI4P 5-kinase *α*. This kinase is responsible for PIP_2_ production from PI4P^45^. In addition, PIP_2_ is metabolised to PIP_3_. This in turn stimulates the small GTPase ARF. The production of PI4P from PI depends on PI4K activity effected by ARF^46^. On account of this positive feedback loop, only a small amount of PIP_2_ is already sufficient to significantly enhance the PIP_2_ level in micromanipulated cells.

The presence of PIP_2_ and phosphorylation of threonine 567 is necessary for ezrin activation. In previous studies we found that PIP_2_ recognition plays a pivotal role in the first step of activation to switch dormant ezrin into the active actin-binding conformation, whereas phosphorylation of Thr-567 is only secondary^17^,^36^. Against this background, the increased level of ezrin co-localised with PIP_2_ in the plasma membrane can be explained. Along the same lines, Shibazaki *et al.* recently reported that actin polymerisation in COS-7 cells increases with increasing PIP_2_ concentration^47^. This is in good accordance with our findings for MDCK II cells.

Based on these results we expected significant changes in the mechanical behaviour of PIP_2_ micromanipulated cells. Most importantly, the tether rupture force *F*_tether_ that is proportional to the square root of membrane tension increases by a factor of two after injection of PIP_2_ micelles into the cell. It is save to assume that the increased membrane tension results nearly exclusively from the fortified connection between the plasma membrane and the underlying actin cytoskeleton. Raucher *et al.* also measured a higher energy necessary for membrane-cytoskeleton separation in fibroblasts containing high PIP_2_ levels using optical tweezers^48^. Accordingly, we found in a previous study that membrane tension drops significantly after disruption of the actin cortex by administration of depolymerising cytochalasin D^27^.

Our indentation experiments show the overall tension *t*_0_ being the sum of membrane tension and cortical tension as well as apparent area compressibility modulus 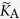 initially increase substantially but are largely restored 5 h after injection of PIP_2_. The increase in *t*_0_ can only partly be explained by an increase in membrane tension as described in our previous study^17^. We attribute this tremendous increase in cortical tension to values, usually found in mesenchymal cells, to a substantial actin remodelling and thereby an increase in actomyosin contractility. There is clear evidence in literature that PIP_2_ regulates actin assembly and also actomyosin contractility^49^,^50^,^51^. Both aspects lead to a higher overall tension. Noteworthy, functions of PIP_2_ go beyond that of ERM protein activation. Among them, production of second messengers, such as PIP_3_ and DAG, membrane targeting, enzyme activation and exo- and endocytosis are the most important and well-established functions of PIP_2_^52^. PIP_2_ enriched rafts might be responsible for enhanced endocytosis thereby explaining an increase in 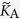. Interestingly, we also found an increase in cortical tension for ezrin depleted cells albeit membrane tension drops since less connections are present between plasma membrane and cortex. We attribute this effect to a change in cellular morphology in response to ezrin depletion. Cells were generally found to display a reduced height by 1–2 μm compared to untreated cells regardless of the treatment chosen to reduce the ezrin-based connection to the plasma membrane. This might be due to loss of polarity and higher contraction of the ezrin-deprived cells. In contrast, cells subjected to PIP_2_ microinjection do not show a change in the overall morphology and therefore the increase in the apparent area compressibility modulus 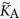 can be largely explained by a change of the accessible surface area. Because of a higher number of connection sites between plasma membrane and cytoskeleton existing membrane reservoirs might be unavailable for buffering stress upon lateral stretching of the membrane. As far as the recovery after 5 h after injection of PIP_2_ micelles is concerned, it is conceivable that other ERM proteins such as moesin, for instance, might substitute ezrin. Interestingly, even an entire removal of microvilli by adding methyl-β-cyclodextrin (MBCD) to extract cholesterol from the plasma membrane results in recovery of mechanical and electrical parameters within a few hours after end of treatment^27^.

**Figure 8:**
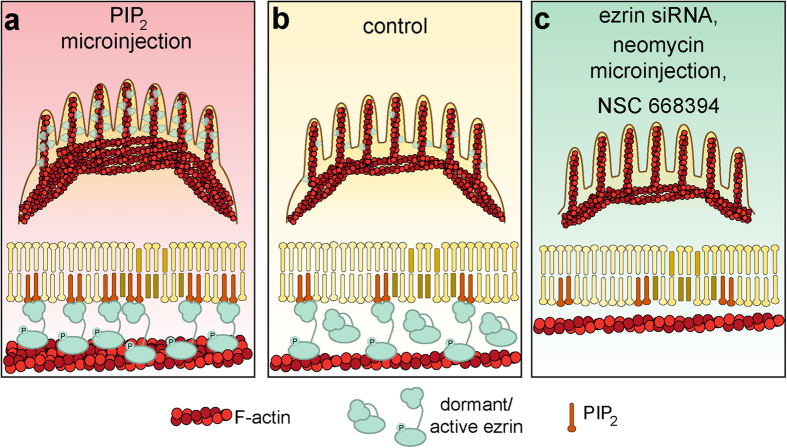
Scheme summarizing the effects on MDCK II cells caused by PIP_2_-microinjection and ezrin depletion. Enhancing PIP_2_ in the inner membrane leaflet leads to a fortification of the membrane-cytoskeleton shell mediated through ezrin. A redistribution of the actin cytoskeleton occurs in response to an increased PIP_2_ level (**a**). Ezrin depletion via ezrin siRNA, neomycin microinjection or ezrin inhibitor NSC 668394, however, results in more condensed, flattened cells (**c**).

In conclusion, we found that epithelial cells regulate membrane tension through ezrin-mediated connections between the inner leaflet of the plasma membrane and the actomyosin cortex (Fig. 8). The PIP_2_ level in the apical plasma membrane controls the number of activated ezrin molecules, which mediate connections between the plasma membrane and underlying cytoskeleton. This is important since an excess of dormant ezrin is located in the cytoplasm and activation of ezrin occurs after binding to PIP_2_ in the inner leaflet through a conformational switch leading to more actin filaments linked to the plasma membrane. Actin remodelling due to a larger amount of PIP_2_ further fortifies cells and thereby renders them extremely stiff and more contractile compared with control cells. Interestingly, the cells largely restore the initial mechanical parameters 5 hours after treatment emphasising the important role of tension homeostasis in epithelial cells. If, however, the number of contacts between the plasma membrane and the actomyosin cortex is reduced, membrane tension decreases and excess membrane surface area disappears, while contractile forces of the cortex increase leading eventually to a collapse of the cells (Fig. 8).

## Methods

### Cell Culture

Madin-Darby canine kidney cells (strain II, MDCK II; Health Protection Agency, Salisbury, UK) were maintained in minimum essential medium (MEM) with Earle’s salts and 2.2 g/L NaHCO_3_ supplemented with 4 mm l-glutamine and 10% fetal calf serum at 37 °C in a 5% CO_2_ humidified incubator. Cells were grown to confluency, released from culture flasks using trypsin/EDTA (0.05%/0.02%) and subcultured weekly. Medium additionally contained penicillin (0.2 mg/mL), streptomycin (0.2 mg/mL) and HEPES (15 μm) during experimentation.

For ezrin inhibition experiments with ezrin inhibitor NSC 668394, cells were seeded on Petri dishes (μ-Dish^35 mm^ Grid-500; ibidi, Martinsried, Germany) and grown to confluency. NSC 668394 (Merck Millipore, Molsheim, France) was dissolved in DMSO. An appropiate amount of this stock solution was added to cell culture media (final concentration: *c*_NSC 668394_ = 250 μm) and cells were incubated for 3 h. For control measurements cells were incubated for 3 h with media containing the same amount of DMSO without NSC 668394.

### Microinjection of PIP_2_ micelles into confluent MDCK II Cells

The microinjection system consists of a microinjector (FemtoJet®; Eppendorf, Hamburg, Germany) and a micromanipulator (InjectMan® NI2; Eppendorf, Hamburg, Germany) equipped with commercially available glass capillaries mounted on an inverted microscope device to allow for phase-contrast microscopy and epifluorescence microscopy. Cells were seeded on Petri dishes (μ-Dish^35mm^ Grid-500; ibidi, Martinsried, Germany), grown to confluency and maintained at 37 °C during the experiment. PIP_2_ from pork brain (Avanti Polar Lipids, Alabaster, USA) was injected together with a metabolic stabilized analogue of PIP_2_ (PIP_2_ *α*-fluorophosphonate; Echelon Biosciences, Salt Lake City, USA) and fluorescein isothiocyanate-dextran (FITC-dextran, MW = 70,000, final concentration: 5 mg/mL) serving as fluorescence marker to identify successfully manipulated cells. All three substances were mixed (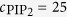 μm in Dulbecco’s phosphate buffered saline without Ca^2+^ and Mg^2+^(PBS^−^)) and homogenized in an ultrasonic bath (50 W, 0.4 s, 30 min) yielding PIP_2_ micelles with an average hydrodynamic radius of 13 nm as determined by dynamic light scattering (ALV/CGS-3 Goniometer System; ALV, Langen, Germany) using a 22 mW He-Ne-laser (*λ* = 632.8 nm; JDS Uniphase, Eningen, Germany). After microinjection of PIP_2_ micelles membrane integrity was verified by using propidium iodide (Vybrant® Apoptosis Assay Kit #4; Life Technologies, Carlsbad, USA) following the manufacturer’s instructions.

For control measurements FITC-Dextran (MW = 70,000, 5 mg/mL in PBS^−^) was injected into confluent MDCK II cells. AFM experiments were performed 3 h after microinjection.

### Ezrin Silencing

MDCK II cells were seeded on Petri dishes (μ-Dish^35 mm^ Grid-500; ibidi, Martinsried, Germany) and grown to 50% confluence. Pooled siRNA targeting ezrin sequences GCUVAAGAUAAUGCUAUGUU, GGCAACAGCUGGAAACAGAUU, GAAGAA-GGCACCUGACUUUUU, and GAUCAG-GUGGUAAAGACUAUU (siGENOME SMARTpool human EZR siRNA; Thermo Fisher Scientific, Lafayette, USA) were transfected using Lipofectamine® RNAiMAX transfection reagent (Life Technologies, Carlsbad, USA) according to the manufacturer’s instructions. Experiments were performed 72 h after incubation with siRNA.

For control measurements non-tageting siRNA sequences UAAGGCUAUGAAGAGAUAC, AUGUAUUGGCCUGUAUUAG, AUGAACGUGAAUUGCUCAA, UGGUUUACAUGUCGACUAA (siGENOME non-targeting siRNA Pool #2, GE Healthcare, Lafayette, USA) were applied.

### Cell Labelling

Cells were grown onto Petri dishes (μ-Dish^35 mm^ Grid-500; ibidi, Martinsried, Germany) to confluency, and fixed with 4% paraformaldehyde in PBS^−^ for 20 min. To block unspecific binding sites and to permeabilize the plasma membrane, cells were treated with blocking buffer (5% (*w*/*v*) bovine serum albumin (BSA), 0.3% (*v*/*v*) Triton X-100 in PBS^−^) for 30 min. For PIP_2_ and ezrin labeling the primary antibody (PIP_2_: mAb mouse IgG2b (Enzo Life Sciences, Lausen, Switzerland), ezrin: mouse IgG1 (BD Biosciences, Heidelberg, Germany)) was diluted with dilution buffer (1% (*w*/*v*) BSA, 0.3% (*v*/*v*) Triton X-100 in PBS^−^) to a concentration of 25 or 4 μg/mL, respectively, and cells were incubated for 1 h at room temperature. The secondary antibody (Alexa Fluor 488- or Alexa Fluor 546-conjugated goat anti-mouse IgG; Life Technologies, Carlsbad, USA) was diluted with dilution buffer down to a concentration of 5 μg/mL and incubated with the cells for 45 min. F-actin was labeled by incubating permeabilized cells with 165 nm Alexa Fluor 488- or Alexa Fluor 546-phalloidin (Life Technologies, Carlsbad, USA) for 45 min. Cell nuclei were labeled with 4′,6-diamino-2-phenylindole (DAPI), diluted to a concentration of 50 ng/mL, and incubated for 15 min. Between every labeling step, cells were rinsed three times with PBS^−^ for 5 min each on a vibratory plate (75 rpm). Plasma membrane labeling was performed using PKH67 Green Fluorescent Cell Linker Kit (Sigma-Aldrich, Steinheim, Germany) or CellMask™ Orange (Life Technologies, Carlsbad, USA) following the manufacturer’s instructions.

Fluorescence imaging was carried out using a fluorescence microscope (BX51; Olympus, Tokyo, Japan) equipped with a water immersion objective (LUMPLFLN 100XW, NA = 1.0; Olympus, Tokyo, Japan) or a confocal laser scanning microscope (CLSM; LSM 710; Zeiss, Jena, Germany or FluoView1200; Olympus, Tokyo, Japan) using a water immersion objective (W Plan-Apochromat 63x, NA = 1.0; Zeiss, Jena, Germany or oil immersion objective UPLFLN100xO2PH, NA = 1.3; Olympus, Tokyo, Japan).

### Solubilisation, Purification and Western Blot Analysis

Cells were grown to confluency in 6-well cell culture plates. After washing cells three times with PBS^−^, cell lysis was achieved by incubation with radioimmunoprecipitation (RIPA) buffer containing a protease inhibitor cocktail (cOmplete EDTA-free tablets; Roche Diagnostics, Mannheim, Germany) for 5 min at 0 °C. After scraping the plate, the lysate was centrifuged at 8,000 × *g* for 10 min at 4 °C. An adequate amount of the soluble fraction was diluted with SDS-PAGE sample buffer, shook at 85 °C for 5 min at 300 rpm and resolved by 17% SDS-PAGE. Proteins were electrophoretically transferred from the gel to a nitrocellulose membrane (supported nitrocellulose membrane 0.2 μm), and blot was blocked with TBT buffer (10 mm Tris-HCl, 150 mm NaCl, 0.2% (*v*/*v*) Tween 20, pH 7.4) containing 5% (*w*/*v*) lowfat dry milk for 1 h using a vibratory plate. The primary antibody (ezrin mouse IgG1; BD Biosciences, Heidelberg, Germany) was diluted with TBT buffer containing 5% (*w*/*v*) lowfat dry milk to a concentration of 500 ng/mL and incubated with the nitrocellulose membrane overnight at 4 °C. After washing the membrane with TBT buffer three times, it was incubated with the secondary antibody (horseradish peroxidase-conjugated goat anti-mouse IgG; Santa Cruz Biotechnology, Santa Cruz, USA), diluted to a concentration of 500 ng/mL with TBT buffer containing 5% (*w*/*v*) lowfat dry milk, for 1 h at 4 °C. Chemiluminescence was developed by using Clarity™ Western ECL Substrate (Bio-Rad, Muenchen, Germany) and exposure to X-ray film.

### Atomic Force Microscopy for Imaging

Cells were fixed using 2.5% (*v*/*v*) glutardialdehyde (GDA) in PBS^−^ for 20 min prior to AFM imaging using a Nanowizard® II or III atomic force microscope (JPK Instruments, Berlin, Germany). Silicon nitride cantilevers (MLCT, Bruker AFM Probes, Camarillo, USA) with a nominal spring constant of 10 mN/m were applied. The exact spring constant of each individual cantilever was determined using the thermal noise method^53^. The AFM was mounted on an inverted microscope enabling phase contrast and fluorescence microscopy during AFM imaging. Cells were imaged in PBS^−^ at room temperature with a scan rate of 0.2 Hz and processed with software provided by the AFM manufacturer.

### Atomic Force Microscopy for Mechanical Measurements

Force indentation experiments were carried out continuously on a Nanowizard® II or III AFM (JPK Instruments, Berlin, Germany) while scanning laterally across the sample referred to as force mapping. Before use cantilevers (MLCT; Bruker AFM Probes, Camarillo, USA) were plasma cleaned (30 s, Argon) and incubated with PBS^−^ containing 2.5 mg/mL concanavalin A-FITC conjugate (Sigma-Aldrich, Steinheim, Germany) for 1.5 h to establish a strong contact between the indenter and the cell membrane for membrane-tether pulling upon retraction from the cell surface. Afterwards, the cantilever was also calibrated using thermal noise method^53^. Prior to indentation experiments cells were seeded on Petri dishes (μ-Dish^35 mm^ Grid-500; ibidi, Martinsried, Germany) and manipulated as desired. After cells reached confluency Petri dishes were mounted on an inverted microscope and kept at 37 °C. Cells were indented up to a force of 1 nN. After a dwell time of 0.5 s the tip was retracted from the cell surface pulling out membrane nanotubes (tethers) from the plasma membrane. The pulling velocity was set to 2 μm/s. Indentation curves were analysed by applying an extended tension model^27^,^39^ (*vide supra*) while tether forces were determined directly from retraction curves (Fig. 1b).

### ECIS experiments

ECIS experiments have been conducted with a homebuilt setup consisting of a SR830 Lock-in amplifier (Stanford Research Systems, Sunnyvale, CA, USA) equipped with a SR550 pre-amplifier (Stanford Research Systems, Sunnyvale, CA, USA). The setup was configured as a voltage divider with a constant ohmic resistor *R*_1_ (*R*_1_ = 1 MΩ) and the measured complex impedance *Z* of the ECIS well. The reference output of the lock-in amplifier was used as voltage source. The resulting frequency dependent in- and out-of-phase voltages were measured by the lock-in amplifier and translated into the complex impedance *Z* of the ECIS well according to


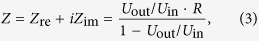


where *U*_out_ = 100 mV is the output voltage of the voltage source and *U*_in_ describes the measured in- and out-of-phase voltages. 20, logarithmically spaced frequencies from 10 to 100,000 Hz were measured. To switch between the different wells of the used 8W1E arrays (Applied Biophysics, Troy, NY) an 8-channel USB-relay (K8090, Velleman, Gavere, Belgium) was used. A self-written Matlab-software was used for data acquisition.

#### Measurement

The 8W1E electrode array (Applied Biophysics, Troy, NY) was placed in a 5% CO_2_ humidified incubator set to 37 °C. 100 μl of cell culture medium were pipetted into each well. 1 h upon addition of the cell culture medium, 200,000 cells suspended in 100 μL cell culture medium were added in each well and incubated for approximately 25 hours. Afterwards, cells were treated for 3 h with 250 μm NSC 668394. Then, the medium containing the inhibitor was completely exchanged by pre-warmed cell culture medium to follow recovery of the cells.

#### Analysis

The ECIS model described earlier by Lo and Ferrier was fitted to the absolute impedance spectra (magnitude 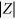 (*ω*)) yielding the barrier resistance of the cell monolayer *R*_b_, the membrane capacitance *C*_m_ and the parameter *α* describing the current flow in the narrow cleft between cell and electrode^54^. *α* is inversely proportional to the square root of the distance between cell and electrode. The results of the fit of each well were normalised to the mean value of the fitting results obtained from the last hour before treatment. In the box plots, the 0 h samples demonstrate the last hour before treatment pooled from three measurements. The 3 h samples demonstrate the normalised results obtained after 2 to 3 hours of incubation pooled together from three independent measurements.

## Additional Information

**How to cite this article**: Rouven Brückner, B. *et al.* Ezrin is a Major Regulator of Membrane Tension in Epithelial Cells. *Sci. Rep.* **5**, 14700; doi: 10.1038/srep14700 (2015).

## Supplementary Material

Supplementary Information
