# Supplementary material for: Ezrin is a Major Regulator of Membrane Tension in Epithelial Cells
Source: Sci Rep. 2015 Oct 5;5:14700. doi: 10.1038/srep14700 (PMC4592969; doi:10.1038/srep14700)
Supplement: Supplementary Information [file srep14700-s1.pdf]

Ezrin is a Major Regulator of Membrane Tension in Epithelial Cells

Bastian Rouven Brückner[1], Anna Pietuch[1], Stefan Nehls[1],
Jan Rother[1], and Andreas Janshoff[1*]

[1]Institute of Physical Chemistry, University of Goettingen,
Tammannstr. 6, 37077 Goettingen

*Correspondence and requests for material should be addressed to
A.J. (email: ajansho@gwdg.de, tel: +49 551 39-10633).

Supplementary Information

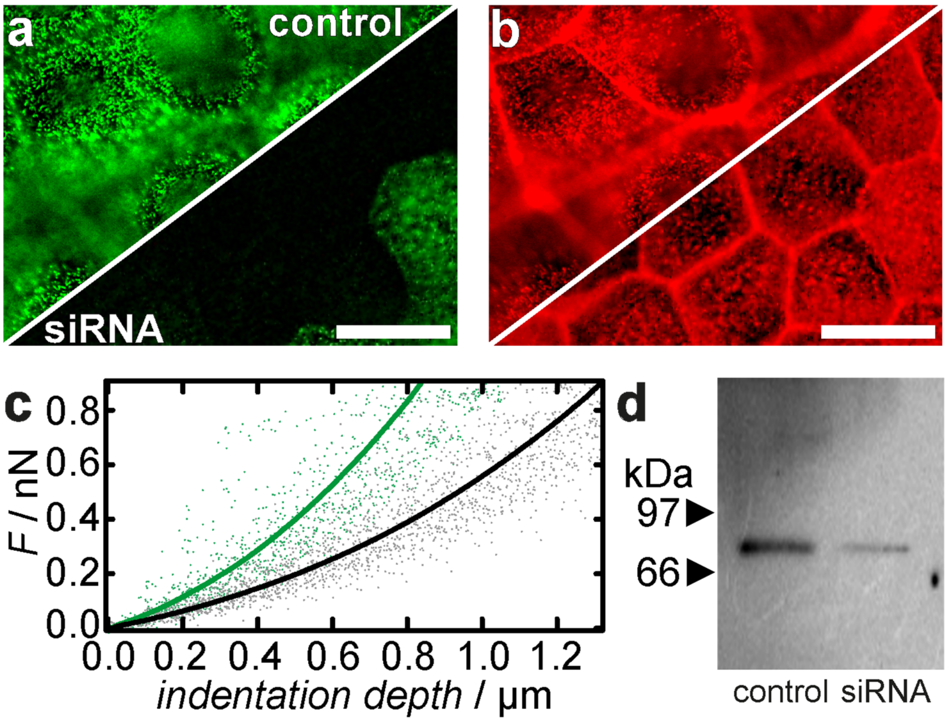

Supplementary Figure S1| Gene silencing for ezrin in MDCK II cells. (**a**) Fluorescence micrograph of ezrin-labelled cells using ezrin mouse IgG primary and Alexa Fluor 488 labelled goat anti-mouse IgG secondary antibody. (**b**) Fluorescence micrograph showing F-actin (Alexa Fluor 546-phalloidin) corresponding to the same spot shown in (**a**). The focus was set to the apical side of the cell. (**c**) Averaged indentation curves for ezrin lacking (green) and non-transfected cells (black). (**d**) Western blot for ezrin. (Scale bar: 20 µm).

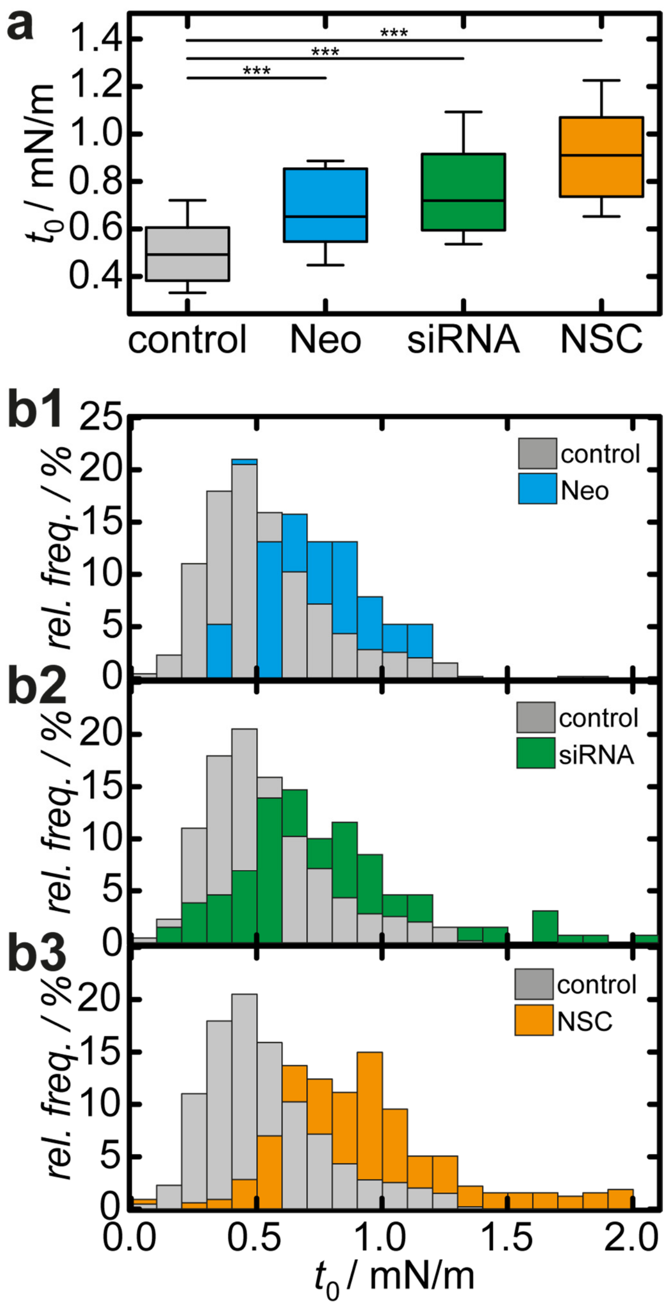

Supplementary Figure S2| Overall tension t_0 of MDCK II cells after ezrin depletion. (**a**) Box plot. (**b**) Histograms showing overall tension after neomycin microinjection (**b1**), siRNA (**b2**) and NSC treatment (**b3**), respectively. Box plots extend from the 30th to the 70th percentile, whiskers from the 20th to the 80th. Grey box plots and bars in the histograms show results from untreated cells, blue ones from neomycin microinjection experiments, green ones represent cells after siRNA treatment, orange ones show results from cells exposed to ezrin inhibitor NSC 668394. n = 389 (control), 38 (neomycin), 129 (siRNA), 317 (NSC 668394) analysed force distance curves. n > 20 (control), n = 2 (neomycin), n > 20 (siRNA), n > 15 (NSC 668394) analysed cells. Asterisks indicate a statistical difference (***: $p < 0.001$, Wilcoxon rank sum test).

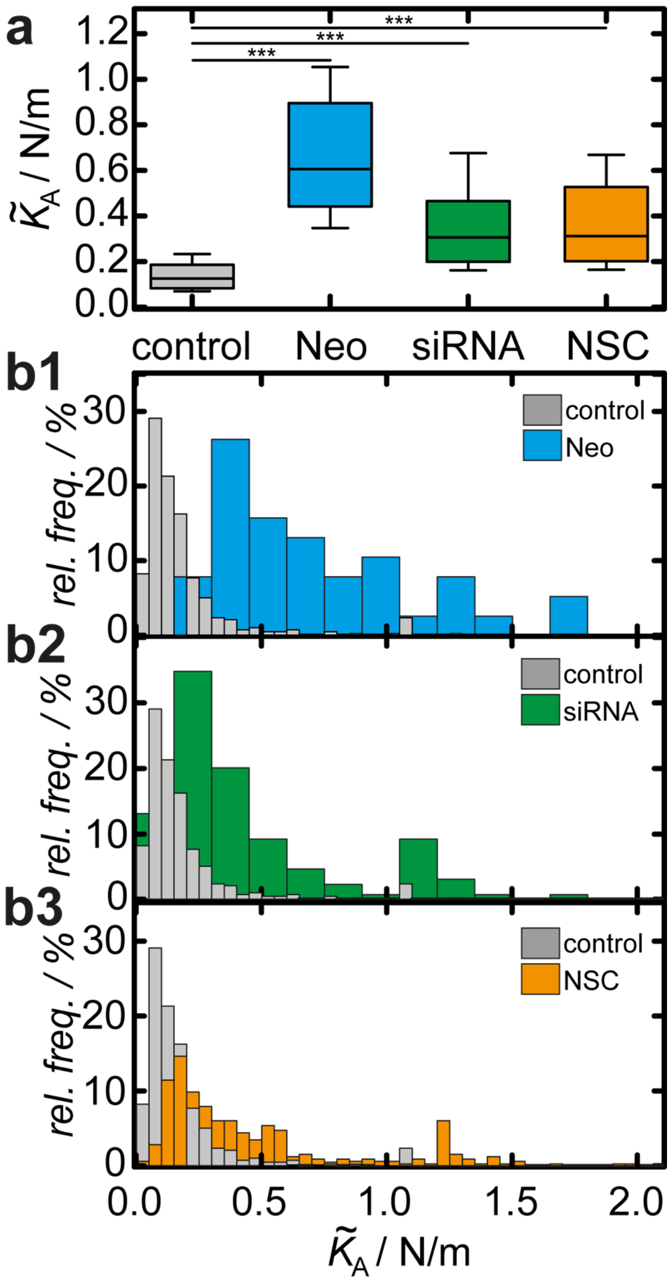

Supplementary Figure S3| Apparent area compressibility modulus $\tilde{K}_A$ of MDCK II cells after ezrin depletion. (**a**) Box plot. (**b**) Histograms showing $\tilde{K}_A$ after Neomycin microinjection (**b1**), siRNA (**b2**) and NSC treatment (**b3**), respectively. Box plots extend from the 30th to the 70th percentile, whiskers from the 20th to the 80th. Grey box plots and bars in the histograms show results from untreated cells, blue ones from neomycin microinjection experiments, green ones represent cells after siRNA treatment, orange ones show results from cells exposed to ezrin inhibitor NSC 668394. n = 374 (control), 38 (neomycin), 129 (siRNA), 313 (NSC 668394) analysed force distance curves. $n > 20$ (control), $n = 2$ (neomycin), $n > 20$ (siRNA), $n > 15$ (NSC 668394) analysed cells. Asterisks indicate a statistical difference (***: $p < 0.001$, Wilcoxon rank sum test).

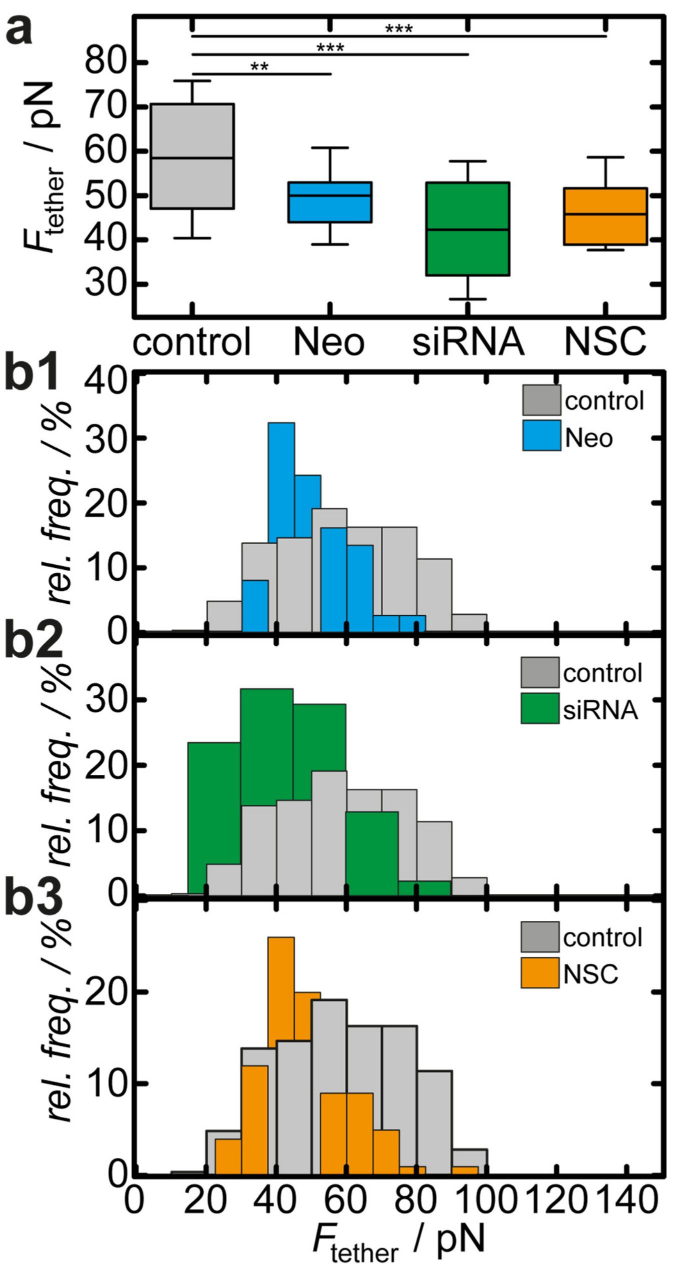

Supplementary Figure S4| Tether rupture force F_{tether} of MDCK II cells after ezrin depletion. (**a**) Box plot. (**b**) Histograms showing tether rupture force after Neomycin microinjection (**b1**), siRNA (**b2**) and NSC treatment (**b3**), respectively. Box plots extend from the 30th to the 70th percentile, whisker from the 20th to the 80th. Grey box plots and bars in the histograms show results from untreated cells, blue ones from neomycin microinjection experiments, green ones represent cells after siRNA treatment, orange ones show results from cells exposed to ezrin inhibitor NSC 668394. $n = 257$ (control), 37 (neomycin), 85 (siRNA), 39 (NSC 668394) analysed force distance curves. $n > 20$ (control), $n = 2$ (neomycin), $n > 20$ (siRNA), $n > 15$ (NSC 668394) analysed cells. Asterisks indicate a statistical difference (**: $p < 0.01$, ***: $p < 0.001$, Wilcoxon rank sum test).

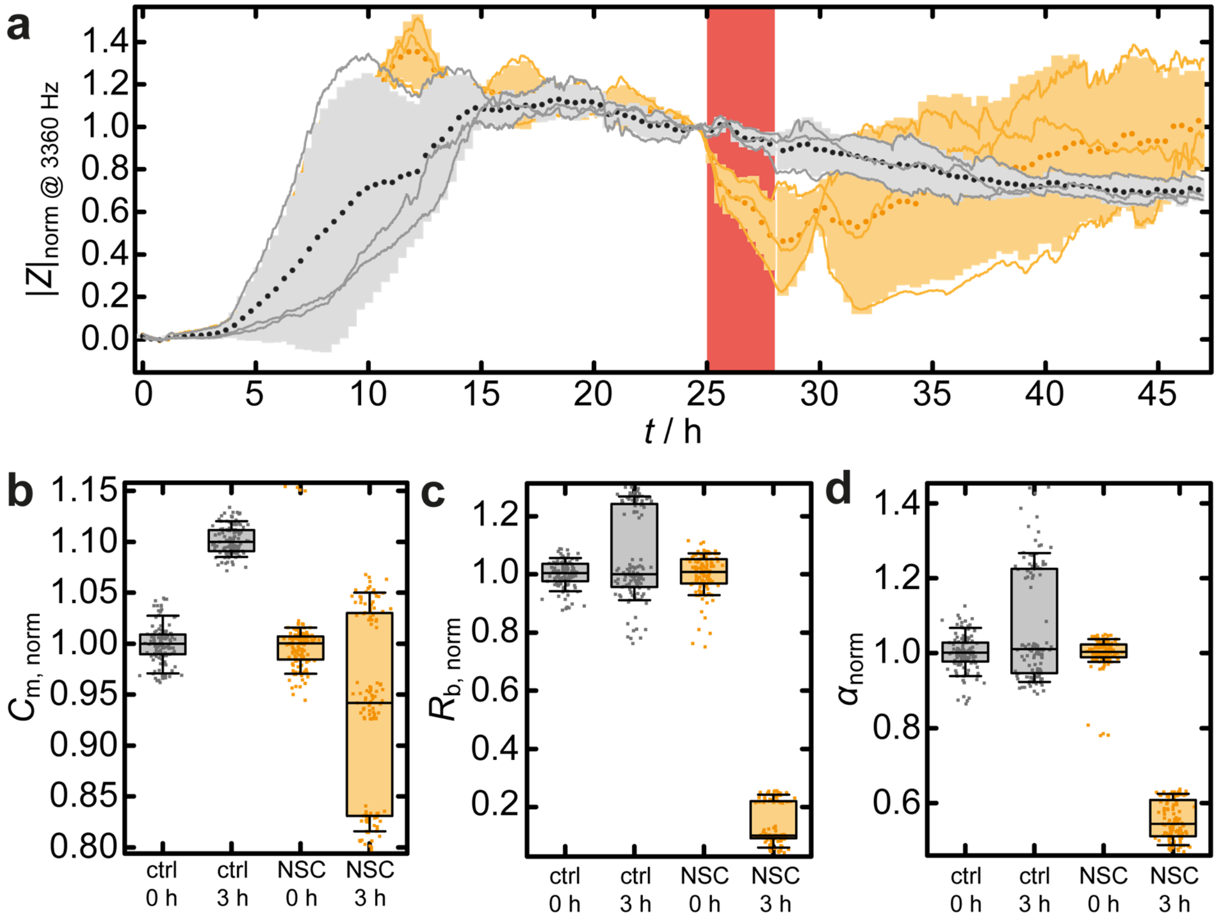

Supplementary Figure S5| ECIS measurements of NSC 668394 treated cells. (**a**) Normalized impedance spectra at 3360 Hz over time. Grey curves represent impedance of untreated cells (—) and the averaged spectra (•), orange ones NSC 668394 treated cells. The incubation time is highlighted in red. (**b**)-(**d**) Box plots showing normalized membrane capacitance C_m (**b**), barrier resistance R_b (**c**) and parameter α describing the current flow between cell and electrode (**d**). Electrode response for control measurements are shown in grey, electrodes for NSC treatment in orange. 0 h samples indicate values obtained from the impedance spectra recorded in a time period of 1 h before addition of NSC, 3 h samples represent values obtained 2-3 h after NSC administration.

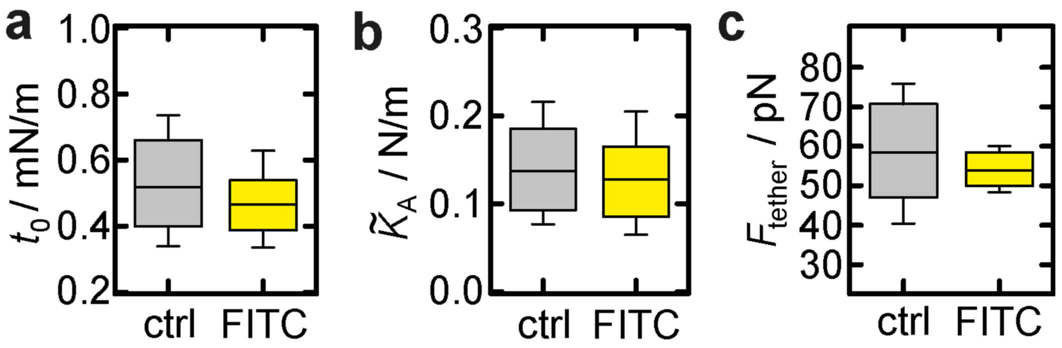

Supplementary Figure S6| Mechanical properties of MDCK II cells after FITC-dextran microinjection. (**a**) Overall tension t_0 obtained from indentation experiments. (**b**) Apparent area compressibility modulus $\tilde{K}_A$. (**c**) Tether rupture force F_{tether} obtained from tether pulling experiments. Box plots extend from the 30th to the 70th percentile, whisker from the 20th to the 80th. Grey boxes represent results from untreated cells, yellow ones are from FITC-dextran microinjection experiments. (**a**) n = 196 (control), 112 (FITC-dextran) analysed force distance curves. (**b**) n = 196 (control), 141 (FITC-dextran) analysed force distance curves. (**c**) n = 257 (control), 81 (FITC-dextran) analysed force distance curves. n > 20 (control), n = 2 (FITC-dextran) analysed cells.

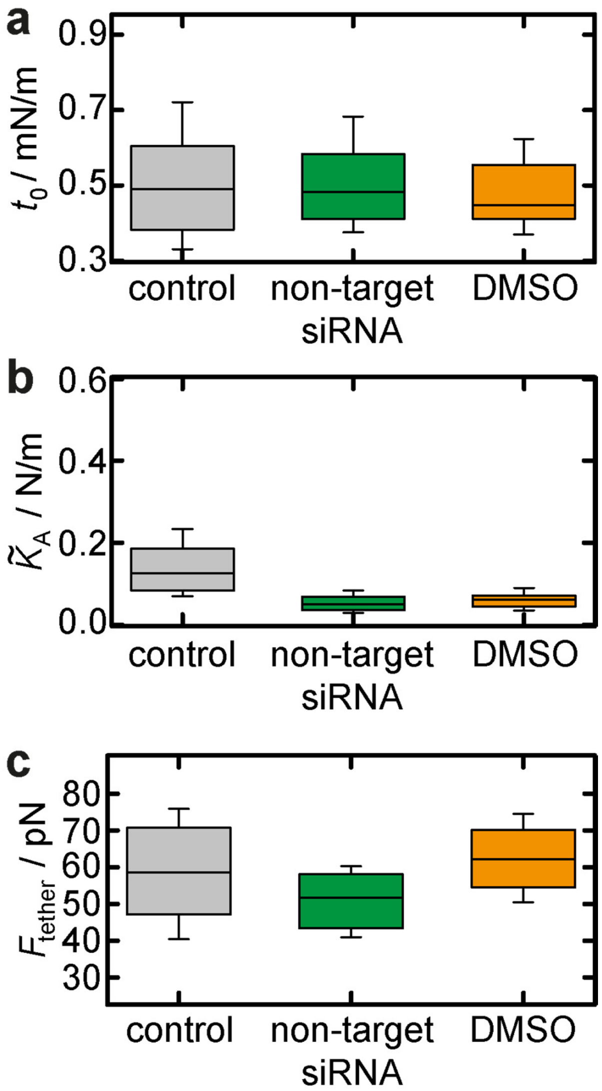

Supplementary Figure S7| Control measurements of MDCK II cells. (**a**) Overall tension t_0 obtained from indentation experiments. (**b**) Apparent area compressibility modulus $\tilde{K}_A$. (**c**) Tether rupture force F_{tether} obtained from tether pulling experiments. Box plots extend from the 30th to the 70th percentile, whisker from the 20th to the 80th. Grey boxes represent results from untreated cells, green ones represent cells after non-targeting siRNA treatment, orange ones show results from cells exposed to DMSO. (**a**) n = 389 (control), 265 (non-targeting siRNA), 62 (DMSO) analysed force distance curves. (**b**) n = 374 (control), 264 (non-targeting siRNA), 61 (DMSO) analysed force distance curves. (**c**) n = 257 (control), 218 (non-targeting siRNA), 228 (DMSO) analysed force distance curves. n > 20 (control), n > 15 (non-targeting siRNA), n > 5 (DMSO) analysed cells.
